# Post-inflammatory Hyperpigmentation in an African American Female: An Atypical Presentation and Treatment Dilemma

**DOI:** 10.7759/cureus.38142

**Published:** 2023-04-26

**Authors:** Sarah A Pearson, Justin D Mark, Jack Bayer, Aaiz Hussain, Hasnan M Ijaz, Muntaha Asif, Mustafa Rahim

**Affiliations:** 1 Internal Medicine, Lincoln Memorial University - DeBusk College of Osteopathic Medicine, Harrogate, USA; 2 Internal Medicine, Dr. Kiran C. Patel College of Allopathic Medicine, Nova Southeastern University, Fort Lauderdale, USA; 3 Internal Medicine, Hospital Corporation of America (HCA) Florida Westside Hospital, Plantation, USA; 4 College of Medicine, Era University, Lucknow, IND; 5 Internal Medicine, Raleigh General Hospital, Beckley, USA

**Keywords:** vitamin d deficiency, autoimmune diseases, functional vitamin b12 deficiency, primary sjogren syndrome (pss), post-inflammatory hyperpigmentation

## Abstract

We present the case of a 32-year-old African American female with a known history of primary Sjogren’s syndrome, multiple vitamin deficiencies, and prior facial cellulitis who presented with diffuse facial post-inflammatory hyperpigmentation following a motor vehicle accident. Following glucocorticoid treatment, only select hyperpigmented areas associated with inflammation, infection, or trauma improved, which thereby posed a clinical challenge to improve the patient’s appearance and condition. Such results may warrant the consideration of adjunctive topical therapies to lighten the remaining areas of hyperpigmentation.

## Introduction

Hyperpigmentation refers to the darkening of the skin due to an increase in melanocytes. Known etiologies of hyperpigmentation include age spots, melasma, and post-inflammatory. Age spots tend to affect advanced-aged individuals and are typically located in sun-exposed skin areas such as the hands and feet. Pregnant individuals and those taking hormonal therapy are the most common patients affected by melasma, which results in large dark spots on the forehead, face, or stomach. Overactivation of melanocytes during an inflammatory process results in post-inflammatory hyperpigmentation (PIH). This overactivation has a higher likelihood of occurring in women with darker skin tones [1]. An increase in melanogenesis results in darker areas of skin after healing. The exact cause of PIH remains unclear, although some studies have linked it to autoimmunity and vitamin deficiencies [2]. While topical therapies such as glucocorticoids, retinoids, and keratolytic agents are often used to treat PIH, the response of patients to these treatments can vary [3]. This case report presents a patient with primary Sjögren's syndrome (SS) and multiple vitamin deficiencies who developed an atypical presentation of PIH following a motor vehicle accident. Despite treatment with glucocorticoids, the patient only had a moderate response, highlighting the challenge of treating PIH.

## Case presentation

A 32-year-old African American woman with a medical history of primary SS and vitamin B12 and vitamin D deficiencies and a remote history of facial cellulitis presented to the clinic after a motor vehicle accident, which resulted in multiple lacerations to her face, excluding her ears. She was taking vitamin B12 and D supplements to correct her comorbid deficiencies. During a physical examination, diffuse discoloration was observed over the forehead and cheeks bilaterally, and dark discoloration was found on the ears bilaterally. Laboratory studies revealed a leukopenic white blood cell (WBC) count of 2.5 x 10^9/L (normal range: 4.5-11.0 x 10^9/L), low vitamin D levels of 8.1 ng/mL (normal range: 30.0-100 ng/mL), low vitamin B12 levels of 3.1 pg/mL (normal range: 232-1235 pg/mL), and positive results for antinuclear antibodies (ANA), antinuclear-ribonucleoprotein (Anti-RNP), anti-Smith antibodies, anti-Sjögren's-syndrome-related antigen A (Anti-SSA), and antichromatin antibodies (Table 1).

**Table 1:** Pertinent laboratory studies revealing leukopenia, multiple vitamin deficiencies, and various positive autoimmune antibody titers. WBC: White blood cell; ANA: antinuclear antibodies; Anti-RNP: antinuclear ribonucleoprotein; Anti-SSA: anti-Sjögren's-syndrome-related antigen A autoantibodies

	Patient	Normal reference
WBC	2.5	4.5-11.0 (x10^9^/L)
Platelets	125,000	150,000 to 450,000
Vitamin D	8.1	30.0-100ng/mL
Vitamin B12	3.1	232-1235 pg/mL
ANA	Direct positive	Negative
Anti-RNP antibodies	8.0	0.0-0.9
Anti-Smith antibodies	2.2	0.0-0.9
Sjogren’s Anti-SSA	1.0	0.0-0.9
Antichromatin antibodies	4.6	0.0-0.9

**Figure 1:**
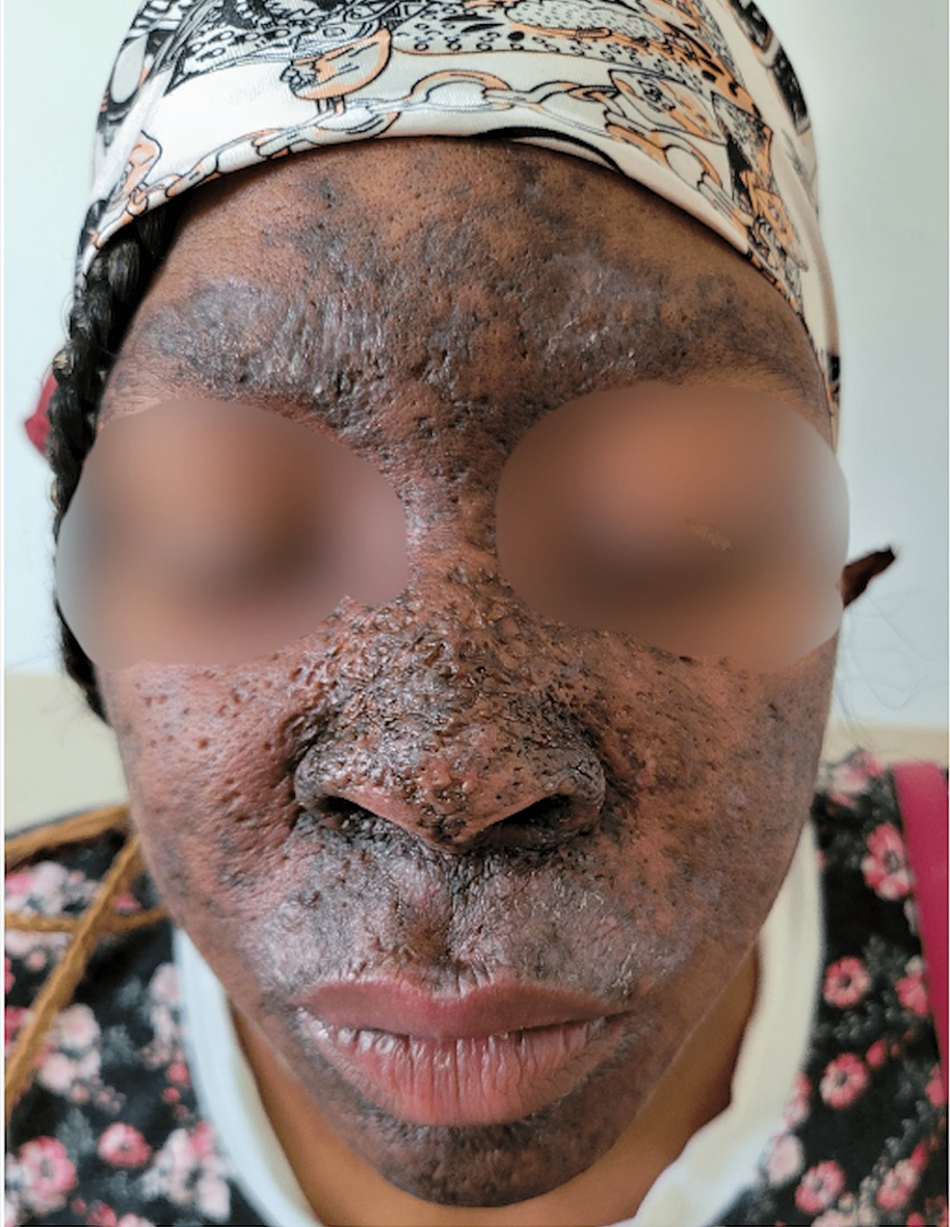
Front view of patient's dermatologic condition prior to treatment initiation Diffuse areas of hyperpigmentation covering near entirety of the face

**Figure 2:**
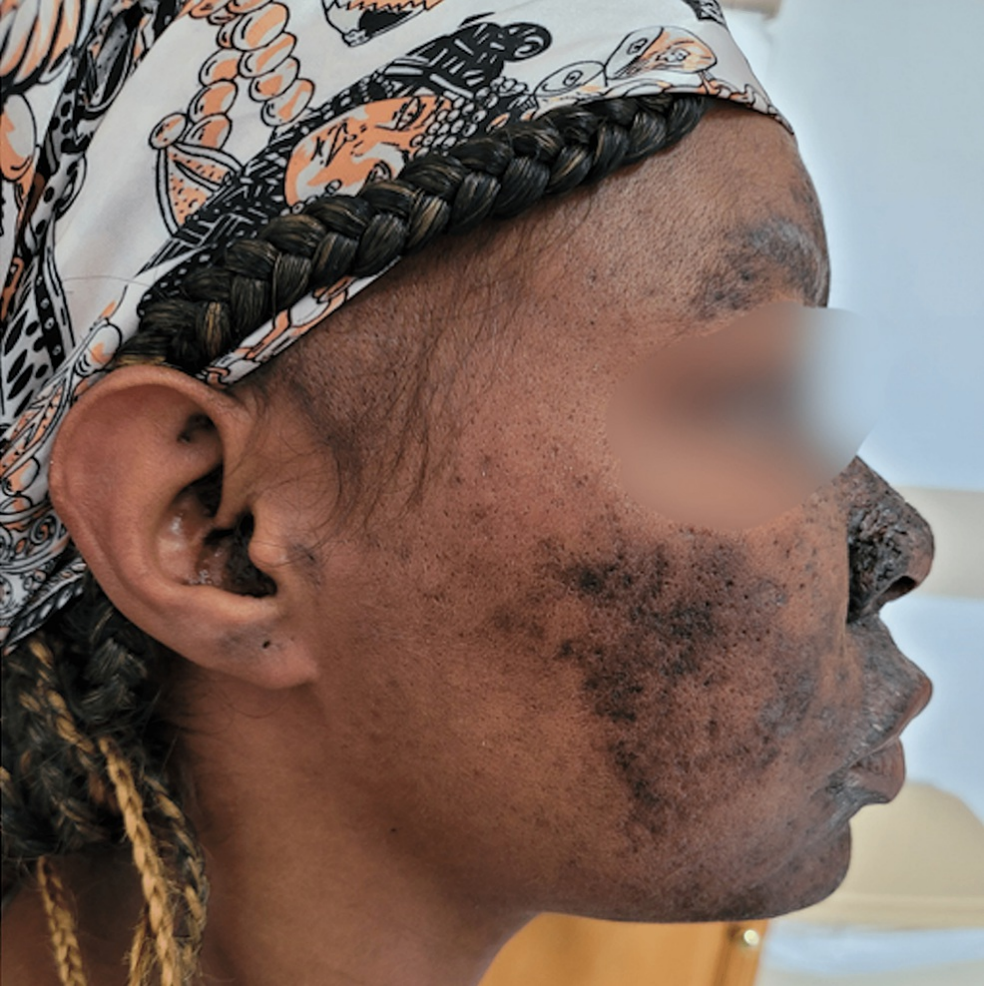
Right-sided view of patient's dermatologic condition prior to treatment initiation Diffuse hyperpigmentation affecting the forehead, nose, and cheek and minor involvement of the ear

The patient expressed concern about the onset of her dermatologic condition. Her concerns were limited to her cosmetic appearance, and she denied any pain or pruritis in the hyperpigmented areas. Photos were taken at the initial visit to utilize for comparison to future visits after the initiation of treatment (Figures 1, 2).

 

**Figure 3:**
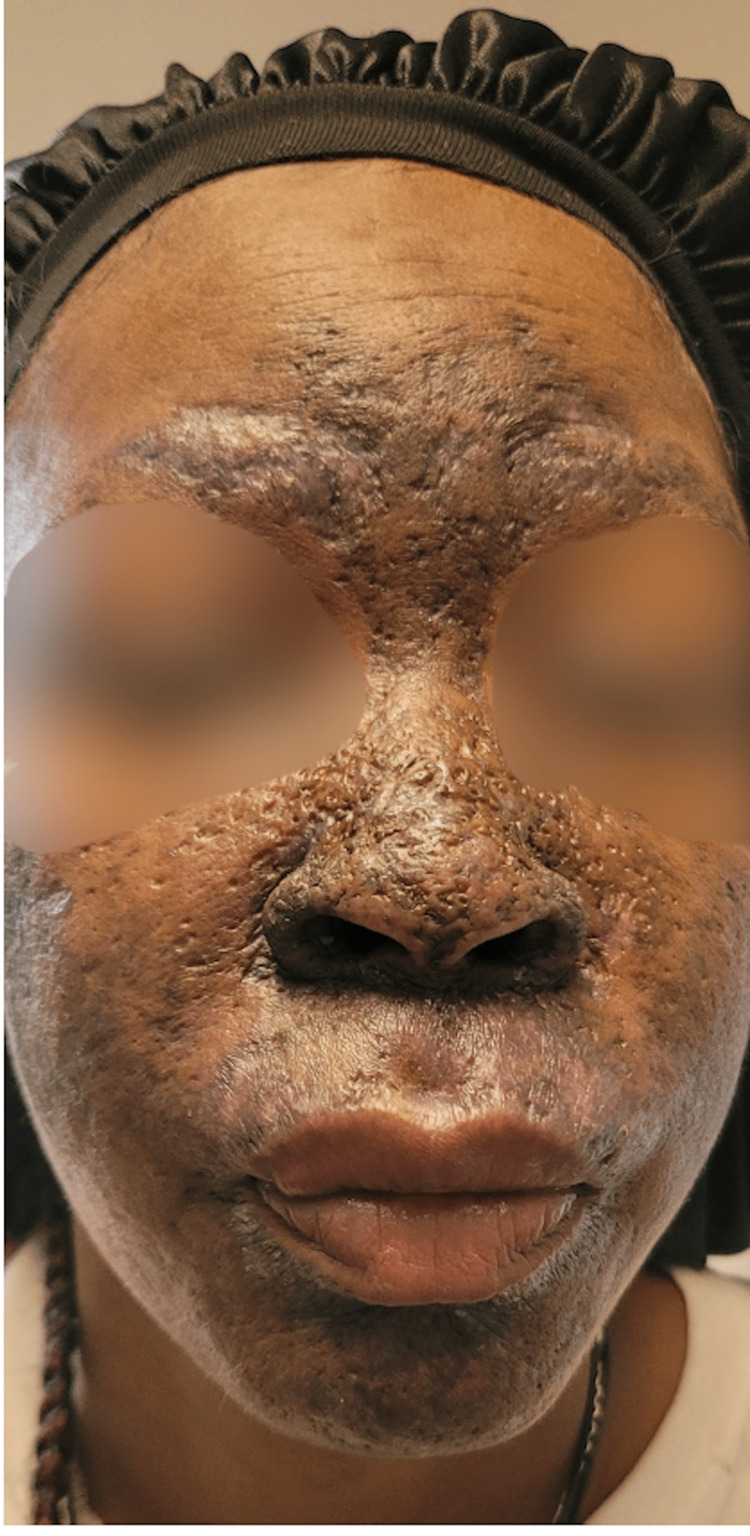
Front view of patient's dermatologic condition five months post-treatment initiation Continued diffuse hyperpigmentation of the face with minimal improvement compared to pre-treatment initiation

**Figure 4:**
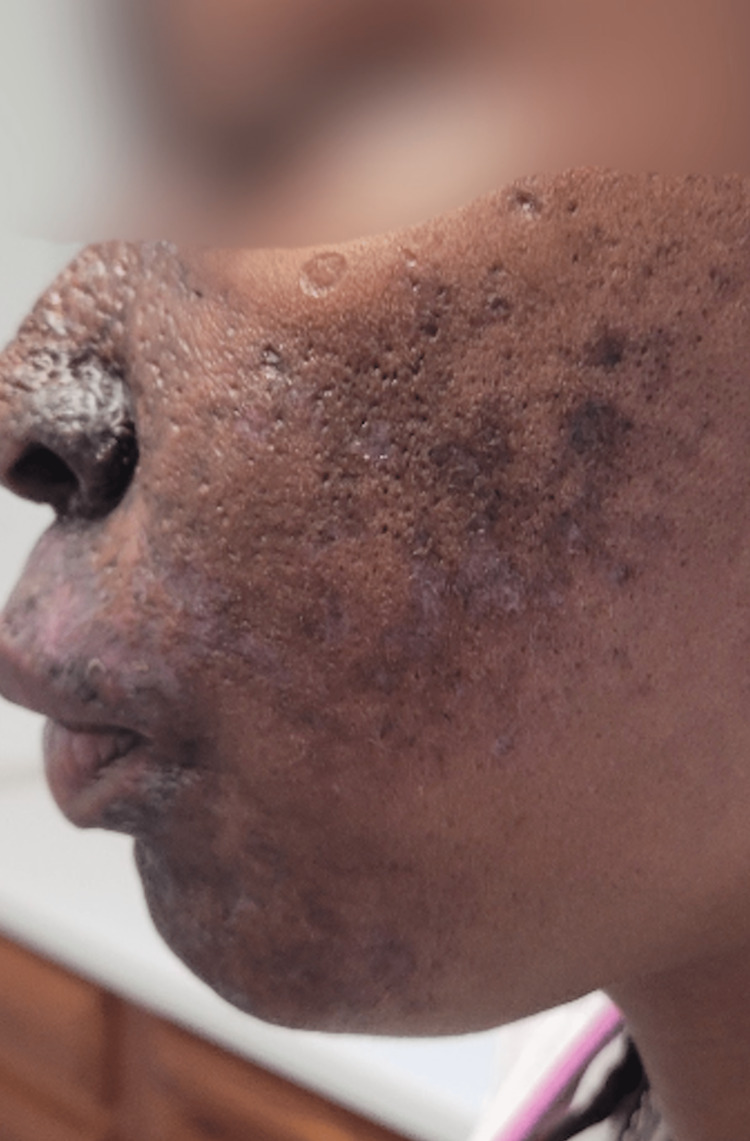
Left-sided view of patient's dermatologic condition five months post-treatment initiation Continued hyperpigmentation with minimal improvement compared to pre-treatment initiation

**Figure 5:**
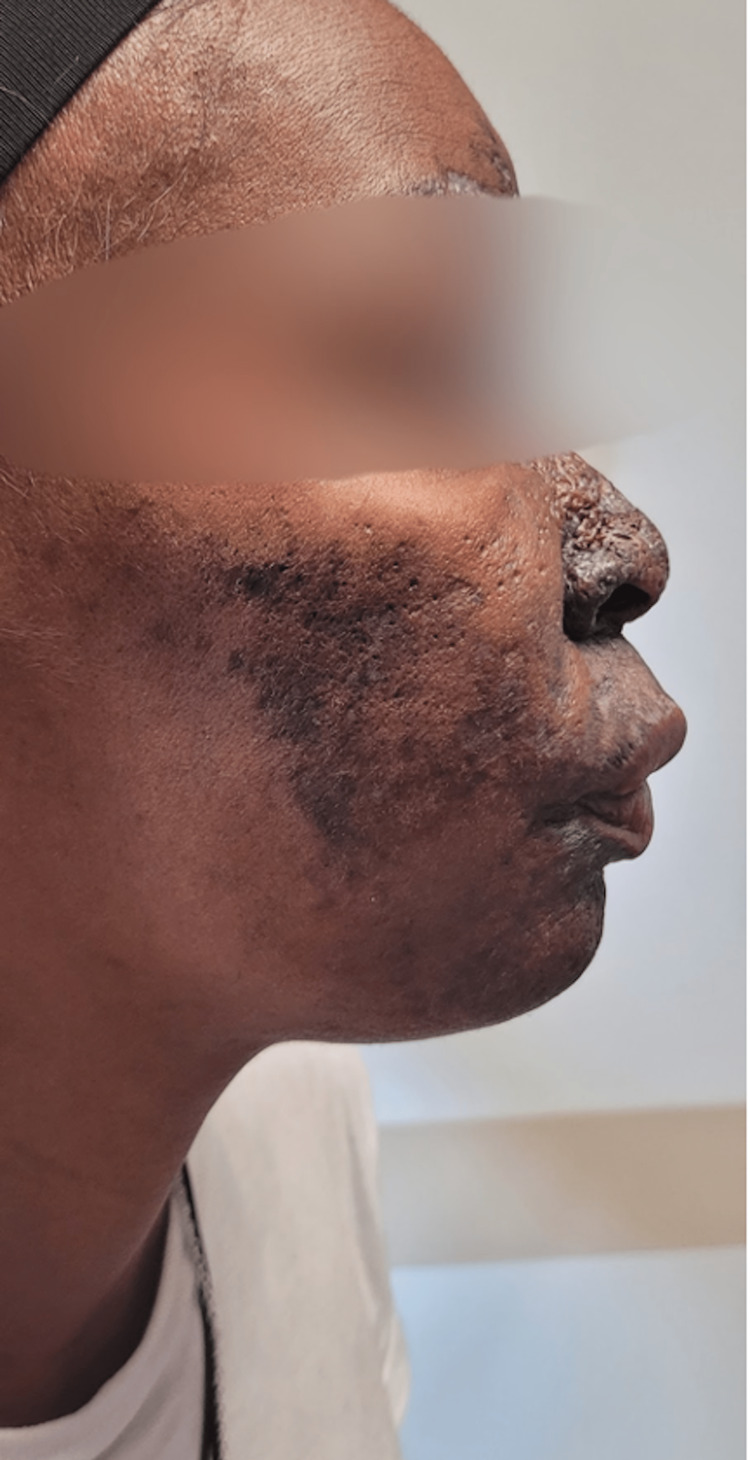
Right-sided view of patient's dermatologic condition five months post-treatment initiation Continued hyperpigmentation with minimal improvement compared to pre-treatment initiation

Corticosteroid treatment was initiated, and she continued to be monitored while instructed to continue her vitamin replacement supplementation. At her five-month follow-up visit, her appearance had minimal improvement, particularly in areas of prior cellulitis and injury (Figures 3-5). While there was some improvement in her condition, the corticosteroid treatment failed to improve the patient's hyperpigmentation affecting her ears, which was not a direct sequela of infection or subsequent hyperpigmentation. 

On follow-up, the patient is currently being monitored for improvement or worsening of her condition.

## Discussion

PIH commonly affects individuals with darker skin tones and those of South American, African, Asian, and Native American descent [4]. Diagnosis of PIH is based on clinical features or Wood’s Lamp examination to distinguish epidermal from dermal PIH. The severity of PIH is determined by the skin type, degree of dermal-epidermal junctional disruption, and inflammation [4].

The exact mechanism of PIH remains unclear, but an increase in melanocyte production occurs within the first week of exposure to an endogenous or exogenous stimulus. Prior autoimmunity, vitamin deficiencies, and trauma are known to trigger this response, which is consistent with the patient's presentation. SS, a common autoimmune disease, has hallmark symptoms of keratoconjunctivitis sicca and xerostomia. Deficiencies in vitamin D and B12 have been implicated in autoimmune conditions such as SS, with B12 deficiency shown to be 39.8% more likely to develop in patients with primary SS than in the general population [5]. Vitamin D is a fat-soluble vitamin essential in bone resorption and calcium levels, and a lack of it can contribute to pigmentation, obesity, and aging. Vitamin D increases melanogenesis and tyrosinase content through its antiapoptotic effect, and normal levels may protect against autoimmunity and hyperpigmentation [6].

Vitamin B12 (cobalamin) is a water-soluble vitamin that plays a role in the metabolism of fatty acids and amino acids and the maintenance of the nervous system. Severe B12 deficiencies present with neuropsychosis and associated symptoms. B12 deficiency has been shown to increase reactive oxygen species (ROS) in melanocytes, which may result in hyperpigmentation. Physiologically, there is a balance between antioxidants and ROS, and deficient levels have been associated with increased ROS in vitro [7].

We investigated the role and mechanism of glucocorticoids and various hormones in treating PIH. Glucocorticoids decrease neutrophil adhesion capability by downregulating both the expression of endothelial adhesion molecules (ELAM-1) and intercellular adhesion molecules (ICAM-1) [8]. The key hormones that play a role in hyperpigmentation are estrogen and progesterone. This is achieved through the membrane-bound steroid hormone receptors G protein-coupled estrogen receptor (GPER) and progestin and adipoQ receptor 7 (PAQR7). These receptors (GPER and PAQR7) can inhibit or promote melanin production, with estrogen as the promontory and progesterone more inhibitory [9].

These results warrant the consideration of adjunctive topical therapies to lighten the remaining areas of hyperpigmentation. Topical depigmenting agents, including retinoids, ascorbic acid, niacinamide, and tyrosinase inhibitors such as hydroquinone, azelaic, and kojic acid, appear effective in mitigating the burden of this condition [10].

## Conclusions

This case report highlights the challenges of treating PIH in patients with primary SS and multiple vitamin deficiencies. PIH is a common condition that affects individuals with darker skin tones and can occur due to endogenous or exogenous stimuli, such as prior autoimmunity, vitamin deficiencies, and trauma. Glucocorticoids, retinoids, and keratolytic agents are commonly used to treat PIH, but their efficacy can vary. Adjunctive topical therapies such as retinoids, ascorbic acid, niacinamide, and tyrosinase inhibitors may be effective in mitigating the burden of this condition. Further research is needed to fully understand the pathophysiology of PIH and develop more effective treatment options.
